# Real and Pseudoaccommodation in Accommodative Lenses

**DOI:** 10.1155/2011/284961

**Published:** 2011-09-18

**Authors:** Ioannis G. Pallikaris, Georgios A. Kontadakis, Dimitra M. Portaliou

**Affiliations:** Institute of Vision and Optics (IVO), University of Crete, Medical School, Heraklion, 71003 Crete, Greece

## Abstract

In the attempt to manage presbyopia, different intraocular lens designs have been proposed such as monofocal IOLs with monovision or multifocal IOLs. Even though the lenses mentioned offer satisfactory visual results, contemporary ophthalmology has not completely answered the presbyopic dilemma by simulating the accommodative properties of the crystalline lens itself. Accommodative IOLs were designed to fill this gap and provide satisfactory vision for all distances by restoring some degree of “pseudoaccommodation.” Pseudo accommodative capability can be linked to monofocal IOL's as well but the results are not satisfactory enough to fully support unaided near vision. Pseudoaccommodation is a complex phenomenon that can be attributed to several static (i.e., pupil size, against-the-rule cylindrical refractive error, multifocality of the cornea) and dynamic (i.e., anterior movement of the implant itself) factors. Objective measurement of the accommodative capability offered by the accommodative IOLs is extremely difficult to obtain, and different methods such as autorefractometers, retinoscopy, and ultrasound imaging during accommodative effort, ray tracing, or pharmacological stimulation have been developed but the results are sometimes inconsistent. Despite the difficulties in measuring accommodation, accommodative IOLs represent the future in the attempt to successfully “cure” presbyopia.

## 1. Introduction

The restoration of near vision in older individuals that have entered the presbyopic age is considered one of the major challenges in refractive surgery during the last decade. There are three principal approaches for the achievement of good near and far vision concomitantly. The first is the establishment of a functional multifocality in the visual system, the second is the establishment of a binocular divergence with one eye focused for far vision and the other for near (i.e., monovision), and the third is the attempt to restore normal accommodation. 

Multifocality and monovision can be established in several ways, surgical or nonsurgical. Nonsurgical ways are with properly designed spectacles and contact lenses. Surgical ways include corneal procedures (i.e., laser surgery on the cornea, thermokeratoplasty, and implantation of corneal inlays) and intraocular procedures that include cataract surgery or refractive lens exchange with the implantation of multifocal intraocular lenses or monofocal lenses with refractive target monovision. The third principal approach for reinstatement of near vision, restoration of accommodation, can be achieved only with surgical ways. Proposed treatments on presbyopic eyes include scleral expansion and femtosecond laser treatment of the lens [1]. Currently the only approach that is clinically applied and has been shown to give promising results is cataract surgery followed by the implantation of accommodating IOLs. 

Accommodative IOLs offer to patients satisfactory near vision by restoring to some degree a dynamic component of the ocular ability for near vision. By implementing several designs of the haptic and the optic part of the IOL, the target is to take advantage of the movement of the ciliary muscle and of the vitreous in order to change position and shape. This offers a change of the overall dioptric power of the eye and the facilitation of near vision. Pseudophakic patients' ability for good distance and near visual acuity without correction can be also noted in patients with monofocal IOLs, and it has been characterized as apparent accommodation or pseudoaccommodation [2]. It has been attributed to several factors such as the pupil size, total and corneal aberrations, degrees and axis of astigmatism, potential of visual perception, and axial movement of the intraocular lens. Axial movement of the IOL is considered to be the dynamic component of this phenomenon, in contrast with the other static components, that is, depth of focus, astigmatism, and so forth. This component of pseudoaccommodation is called pseudophakic accommodation, while the static component is called pseudophakic pseudoaccommodation.

## 2. Ciliary Muscle in Presbyopic Eyes

Considerable amount of data proves that pseudophakic IOLs can respond to ciliary body contraction with axial displacement. The function of accommodative IOLs is based on the concept that ciliary muscle movement is supposed to be preserved during presbyopia progression. There are studies in animal models that show degradation in the ability of the ciliary muscle to offer accommodation with age, but without this being the only reason for the loss in accommodative amplitude [3]. Age-related changes in the ciliary muscle have been described by Pardue and Sivak [4]. These investigators have demonstrated that ciliary muscle of older subjects contained greater amounts of connective tissue, was shorter, wider, and the internal apical edge moved forward but retained the ability to contract. Studies have been conducted in humans with the use of ultrasound and magnetic resonance imaging, in order to demonstrate any changes in the ciliary muscle structure and function related to age and correlated with cataract surgery. Park et al. [5] have shown that, although there is a decrease in contractility of ciliary body with age in phakic patients, after cataract surgery contractility increases. According to Park et al., lenticular sclerosis negatively affects ciliary body contraction capability. Strenk et al. [6] have shown with magnetic resonance imaging that the ciliary muscle remains active throughout life, although there are age-related changes that may interfere with presbyopia. Stachs et al. [7] also demonstrated with the use of ultrasound that the ciliary body is active in the presbyopic age.

## 3. Measurement of Apparent Accommodation

Apparent accommodation has been assessed in several studies by means of several objective and subjective methods. Most of the subjective methods that have been used are dealing with the depth of focus by investigating the nearest point of fixation without subjective blur. Objective methods are comprised of autorefractometers or retinoscopy during accommodative effort. Ray tracing aberrometry has also been utilized for the objective measurement of accommodation, having as a limitation the difficulty to evaluate aberrations in constricted pupils. Due to the high disparity of the methods that are used there is significant inconsistency in the results of many studies [8, 9]. Another source of inconsistency is the fact that several studies in order to evaluate accommodative response of the IOLs use pharmacologic stimulation of accommodation with pilocarpine, or pharmacologic relaxation with cyclopentolate, or both in different time points. It has been demonstrated that pharmacologic stimulation overestimates the accommodative effort, and thus the results of such measurements may be indicative of the potential of accommodative IOLs; the real life conditions are different. 

Similar drawbacks in the literature exist when investigating the potential forward movement of accommodative IOLs [10]. The most commonly used method for this measurement is the evaluation of the anterior chamber depth during relaxation and accommodation. The utilized equipment for these measurements in several studies is ultrasound biomicroscopy, partial coherence interferometry Scheimpflug imaging, and optical coherence tomography and the results of the methods mentioned above are not equivalent. One of the sources of inconsistency between the results of these measurements is the method for stimulation of accommodation utilized in each study. For example in studies utilizing ultrasound, accommodative effort is stimulated by fellow eye stimulation. On the contrary with partial coherence interferometry the eye under examination can view the target that stimulates accommodation. The fellow eye can be occluded thus avoiding convergence movements. Tests are conducted either under normal stimulation of accommodation or under pharmacologic stimulation of accommodation with noncomparable results. Pharmacologic stimulation overestimates forward movement and may not adequately simulate daily life performance of accommodative IOLs. However, it may be helpful in evaluating the maximum potential of an accommodating IOL. Despite that, there are studies that do not confirm pseudoaccommodative capability, showing no movement or even backward movement of the IOLs under these conditions [11, 12].

Evaluation of presbyopia and of the results of presbyopia treatment is often done by means of near vision assessment. Although this is a key part of many studies, there is a lack of standardization of methods for near vision assessment. A large variety of near vision charts are available, with different fonts and font sizes, having as a result noncomparable measurements. Lately new methods have been developed, such as the MNRead Acuity charts that evaluate reading acuity, speed, and critical print size [13] and the UoC logMAR charts for near and intermediate vision that evaluate near visual acuity in the Log MAR scale with letters suitable for European-wide use (Tsilimbaris MK, Plainis S, Tontos C, Kontadakis G, Pallikaris I, Normative Distance, Intermediate And Near Visual Acuity In Simulated Presbyopia, Invest Ophthalmol Vis Sci 2011; 52: E-Abstract 2833). Nevertheless, reading conditions may differ between examination settings, resulting in non-comparable outcomes. Thus methods such as the Salzburg Reading Desk have been developed, in order to asses near vision in standardized conditions of luminance and contrast [14]. Disparity between methods used in different studies remains a significant drawback in evaluation of presbyopia treatment results.

## 4. Pseudoaccommodation in Monofocal IOLs

Although the refractive results of cataract surgery with monofocal IOL implantation are excellent, the concomitant correction of unaided far and near vision is not satisfactory. Implantation of a monofocal IOL results in the elimination of natural accommodation, with a restoration of an optically solid eye, without the ability of focusing in different distances. This results in a necessity for near distance spectacles after cataract surgery when unaided far vision is aimed postoperatively. At the same time, there are patients with good distance and near visual acuity without correction due to apparent accommodation. In most of these cases the apparent accommodation is not sufficient to provide a functional unaided visual acuity, but there are patients that achieve spectacle independency in their everyday activities, because of this phenomenon.

Hayashi et al. [15] demonstrated that near vision ability of pseudophakic patients decreases proportionally with age. Authors speculated that the main causative factor for this phenomenon is the aging decay of visual perception and acknowledged the potential visual perception as a significant parameter for pseudoaccommodation. K. Hayashi and H. Hayashi [16] studied apparent accommodation in pseudophakic eyes and made comparisons with amplitude of accommodation in phakic age-matched subjects. There were significant differences in ages less than 60 years. The amplitude of apparent accommodation was considered virtually equivalent to that of normal accommodation in patients older than 60 years of age.

Depth of focus, an intrinsic characteristic of all optical systems that is attributed to aberrations and pupil size, is also considered to be one of the most significant factors of apparent accommodation. Nakazawa and Ohtsuki [17] demonstrated that the amount of apparent accommodation is highly correlated to the calculated depth of focus in eyes implanted with monofocal IOLs. In contrast, depth of focus is negatively correlated with normal visual acuity [18]. The higher the visual acuity, the lower the depth of focus. Spherical aberration is a significant component of increased depth of focus in the normal human eye. A decrease in total spherical aberration is achieved with aspheric IOL implantation, in order to achieve an optimum visual acuity and visual quality in terms of contrast sensitivity. Studies of near visual acuity in patients with aspheric versus spherical IOLs show controversial results. It has been reported by Rocha et al. [19] and by Nanavaty et al. [20] that patients with aspheric IOLs show lower depth of focus and reduced distance-corrected intermediate and near visual acuity in comparison with patients implanted with spherical IOLs. At the same time there are studies that show no difference in pseudoaccommodation between patients with aspheric and spherical IOLs [21]. In a study by Oshika et al. [22] of the effect of corneal aberrations and multifocality on pseudoaccommodation, the corneal multifocality was found to be highly correlated and also the coma-like aberration, in contrast with spherical aberration which was not correlated to pseudoaccommodation. Another study supporting these results showed a significant positive correlation of apparent accommodation with vertical coma but a negative correlation with spherical aberration [23]. Also the pupillary diameter has been demonstrated to be inversely proportional to pseudoaccommodation [24], as it has a significant effect to the depth of field. 

One of the most significant contributing factors to apparent accommodation seems to be against-the-rule astigmatism [25]. When compared to with-the-rule astigmatism, against-the-rule offers better near visual acuity, while the unaided far vision is equally affected [26]. Verzella and Calossi [27] calculated that a moderate myopic astigmatism of −1.5 D × 90° can often offer pseudophakic patients a rewarding spectacles independence both for distant and near vision. Nanavaty et al. [28] conducted a study comparing a group of patients with good distant and near visual acuity with a control group (both implanted with monofocal IOLs). Several parameters were evaluated and the existence of against-the-rule astigmatism was the only demonstrating statistically significant difference between the groups. Authors speculated that astigmatism attributes to the increased depth of focus, but also it may show significant differences in near visual acuity due to the fact that the Latin alphabet has a significant vertical component, thus being more easily read by patients having against-the-rule astigmatism, that is, having the vertical focal line on the retina during near vision. 

Axial movement of the IOL is a factor that has also been studied as a contributing factor to pseudoaccommodation of monofocal IOLs. According to published equations pseudophakic accommodation depends on four parameters, the range of movement of the IOL, the position of the IOL, the keratometric values, and the axial length [29]. It has been calculated that accommodation obtained per 1.0 mm of forward IOL movement varies with axial length from 0.8 D in a long eye to 2.3 D in a short eye [30].

Several in vivo studies have measured the movement of monofocal IOLs during accommodating effort, in terms of anterior chamber depth difference in far and near fixation of the subjects. Also it has been measured as the difference between anterior chamber depth during pharmacologic stimulation of ciliary body contraction with pilocarpine and during ciliary body relaxation with cycloplegia. The results of these studies are contradictory. When examined under physiologic accommodation, the movement of the monofocal IOLs seems in several studies to be neither significant nor sufficient to provide accommodation [31–35]. On the other hand, in a study of young pseudophakic subjects IOL movement has been shown to be a significant part of pseudoaccommodation [36]. When studied under pharmacologic stimulation of accommodation or of relaxation, most of the studies find a forward movement of the IOL in the vicinity of a few deciles of a millimeter. On the contrary, there are studies that report a backward movement of the IOL under pilocarpine. When compared to normal stimuli-induced movement, pharmacologically induced movement of the IOLs is higher. As a result it is not yet clear whether the movement of the IOL plays a role in pseudoaccommodation, as far as monofocal IOLs are concerned.

## 5. Pseudoaccommodation in Accommodative IOLs

Accommodative IOLs are designed to provide pseudophakic accommodation. Currently available accommodating IOLs are based on the principle of forward movement of the optics. The contraction of the ciliary muscle is supposed to initiate an anterior shift of the lens optic and thus increase the total refractive power of the eye. With implantation of these IOLs, subjective accommodative amplitudes of up to 2.0 diopters (D) and spectacle independence have been reported in most patients. An IOL optic shift of 1.0 mm can offer about 1.0 D of accommodation in a single-optic IOL and 2.5 to 3.0 D in an IOL with 2 lens optics [37, 38]. The amount of accommodative result depends on several factors, such as the refractive power of the IOL and the position of the optics in the capsular bag or the posterior chamber. 

Two of the most studied in the literature accommodative IOLs that are currently clinically available are the Crystalens AT-45 and the 1CU (Human Optics AG). 

The 1CU is a foldable single-piece IOL that has an optic diameter of 5.5 mm and overall length of 9.8 mm. It is of a hydrophilic acrylic with an ultraviolet inhibitor and has a refractive index of 1.46. The lens has a biconvex square-edged optic and 4 modified flexible haptics that are designed in order to bend when constricted by the capsular bag after ciliary muscle contraction. This allows anterior displacement of the optic resulting to refractive power increase.

Most of the studies of the 1CU IOL demonstrate the efficacy of this IOL in restoring near vision and spectacle independency for near vision. Accommodative range with this IOL has been measured subjectively and objectively. Mastropasqua et al. [39] found that mean amplitude of accommodation was 2.36 D from 1 month to 3 months post-surgery and 1.90 D 6 months after surgery and concluded that posterior capsule fibrosis may interfere with the IOL function. Saiki et al. [40] evaluated both subjective and objective accommodative amplitude and found that subjective mean amplitude of subjective accommodation was 2.25 D, 1.33 D, and 1.36 D at 1 month, 1 year, and 4 years, respectively; none of the changes was statistically significant and, they concluded that contraction of the lens capsule might eventually cause lack of accommodation. Despite that objective accommodative amplitude was 0.68 D without any variations over time. Dogru et al. [41] reported peak objective accommodation amplitude of 0.50 D 3 months postoperatively that decreased thereafter. Wolffsohn et al. [42] found mean objective amplitude of 0.72 D 4 months after implantation of the accommodating IOL and mean subjective amplitude of accommodation of 2.2 D. In this study, two years after 1CU implantation, refractive error and distance visual acuity remained relatively stable, but near visual acuity and the subjective and objective amplitudes of accommodation decreased. The authors conclude that the objective accommodating effects of the 1CU lens appear to be limited and the greater subjective amplitude of accommodation is likely to result from the eye's depth of focus. Küchle et al. [43] evaluated amplitude of accommodation by 3 different methods (near point, defocusing, and retinoscopy) in a comparative study of the 1CU with monofocal IOLs. They observed a higher accommodative range with all 3 methods (mean 1.83 versus 1.16 D (near point), 1.85 versus 0.64 D (defocusing), and 0.98 versus 0.17 D (retinoscopy)). Different methods showed different results but the 1CU accommodative IOL showed increased accommodative range and better near visual acuity than a control group with conventional IOLs in all measurement methods. 

The ability for a forward movement during accommodation effort has been studied in detail in the literature. Marchini et al. [44] studied ciliary body contraction and measured movement of the 1CU and monofocal control by means of ultrasound biomicroscopy using a 50 MHz transducer probe (UBM System 840, Carl Zeiss Meditec, Dublin, CA) at 1, 6, and 12 months after surgery. The UBM data show that every operated eye (whatever the implant) reacted by rotating the ciliary body in an anterior direction when accommodation was solicited by a near target. This rotation correlated very well with the IOL forward movement only in those eyes implanted with accommodative IOLs. Authors state that these results support the hypothesis that accommodative IOLs proportionally react to ciliary body rotation. Hancox et al. [45] used a commercially available partial coherent interferometer to assess movement of the 1CU IOL and of a monofocal IOL under accommodation effort and also after pilocarpine instillation. A small anterior movement of 0.010 mm of the 1CU was detected with accommodation. After pilocarpine 4% instillation, a forward movement of 0.220 mm was seen with the 1CU compared to a backward movement of 0.028 mm with the monofocal IOL. They found no significant correlation between distance-corrected near visual acuity and IOL movement. Authors conclude that the amount of the IOL shift was not sufficient to provide useful near vision, but the difference suggests that the engineering concept behind the 1CU IOL is valid. Langenbucher et al. [46] measured anterior chamber depth using the IOL Master before and after instillation of pilocarpine 2% drops in patients implanted with the 1CU IOL and a monofocal IOL. They found a mean forward shift of 0.78 mm in the 1CU group and 0.16 mm in the control group, indicating a calculated accommodation of 1.16 D versus 0.22 D. Findl et al. [47] measured the movement of the 1CU under pilocarpine with partial coherence laser interferometry and found a forward movement under pilocarpine with a mean amplitude of movement of 0.314 mm, compared with the backward movement of 0,063 mm for the monofocal control IOL. The amount of movement was calculated to result in a refractive change of 0.5 diopters (D) in most patients, reaching 1 D or slightly more in only single cases, with a large variability of movement.

Crystalens AT-45 is another accommodative IOL that has been widely studied. It is the first IOL approved by the FDA that provides pseudoaccommodation capability postoperatively. The Crystalens AT-45 is a biconvex lens with a 4.5 mm optic and flexible hinged-plate haptics that allow forward movement of the optic during accommodative effort to provide near and intermediate vision in pseudophakic patients. The lens design incorporates grooves, or hinges, across the plates adjacent to the lens optic that allow for forward and backward movement of the plate-haptic lenses against the vitreous face. The proposed mechanism of the Crystalens AT-45 IOL is that, with accommodative effort, there is a redistribution of the ciliary muscle mass that causes increased vitreous pressure and forward movement of the IOL. 

The results of the FDA trial for this IOL demonstrated that nearly all patients (74 patients; 97.3%) who bilaterally were within 0.50 D or plano-, postoperatively achieved 20/32 (J2) or better uncorrected near, intermediate, and distance visual acuities [48]. The Crystalens AT-45 accommodating IOL provides good uncorrected near, intermediate, and distance vision in pseudophakic patients. Contrast sensitivity with the Crystalens AT-45 was not diminished relative to standard monofocal IOLs, and near and intermediate visual performance was significantly better than with standard IOLs.

Accommodation amplitude with the AT 45 was measured by Marchini et al. [44] with the negative-lens test, in which a negative lens of increasing power (0.25 D steps) was added to the best corrected distance acuity. The maximum additional dioptric value that allows the best corrected distance acuity was considered an indirect measurement of accommodative amplitude. Mean accommodation amplitude was found at 1.09 D at 3 months and 1.08 at 6 months postoperatively. The same study evaluated forward movement of the IOL by means of ultrasound biomicroscopy with 50 MHz transducer probe by evaluating anterior chamber depth during relaxation and stimulation of normal accommodation. During accommodation, the mean reduction in ACD was 0.32 mm at 1 month and 0.33 mm at 6 months. There was a correlation between accommodative amplitude and a decrease in the ACD. Stachs et al. [49] evaluated objectively accommodation amplitude with the AT 45 under pharmacologically induced accommodation. Mean forward shift of 0.13 mm was observed under pilocarpine treatment. Accommodative amplitude of 0.44 D was found using a Hartinger coincidence refractometer. The mechanical performance of the AT-45 in these eyes did not appear to provide the range of accommodation necessary for close work.

Macsai et al. [50] measured accommodation using 1 objective (dynamic retinoscopy) and 2 subjective methods (defocus and near point of accommodation) in patients implanted with the AT-45 and a monofocal IOL. Measures of accommodation were significantly higher in Crystalens patients than in the monofocal IOL patients (dynamic retinoscopy 2.42 D versus 0.91 D monocular defocus 1.74 D versus 0.75 D; monocular near point of accommodation 9.5 inches versus 34.7 inches).

In another study by Marchini et al. [51] accommodation amplitude and IOL forward shift of AT-45 were evaluated 1 month and 12 months postimplantation in comparison with monofocal IOLs and the 1CU. Accommodative amplitude was indirectly calculated by the minus lenses procedure, and IOL movement was evaluated by means of ultrasound biomicroscopy. Accommodation amplitude was not found to differ significantly between the groups although distance corrected near visual acuity was significantly better in AT-45 than in monofocal IOLs. Forward shift of the IOL assessed by means of variation in anterior chamber depth with AT-45 was 0.24 mm at 1 month and 0.17 at 12 months postimplantation, and in monofocal controls there was a slight backward shift. These results support the hypothesis that accommodative IOLs proportionally react to ciliary body rotation. 

A new, improved Crystalens model, the Crystalens HD that has received FDA approval as well, has a mechanism of action that is based on the transitional movement of the lens in anterior and posterior direction due to ciliary muscle contraction and vitreous mass displacement. Alió et al. [52] performed a comparative study of implantation of the Crystalens HD accommodative IOL in one group (16 eyes) in comparison with implantation of a monofocal IOL in a second group (24 eyes). In both groups after cataract surgery uncorrected distance visual acuity was restored successfully. Accommodative IOL showed an advantage with respect to uncorrected near vision restoration while optical quality found no statistically significant differences between the two groups. 

The Tetraflex (model KH3500, Lenstec, Inc.) accommodative IOL is currently under trial for FDA approval. The Tetraflex is a single-piece posterior chamber IOL with flexible 10-degree anteriorly angulated closed-loop haptics and a spherical optic. The hydrophilic IOL can be inserted through a small (2.50 to 3.00 mm) clear corneal incision. Lens design provides flexibility and vaulting allowing anterior movement of the lens and providing satisfactory near vision [53]. An alternative mechanism of action of the Tetraflex described by Wolffsohn et al. [54] is that near vision benefits can be attributed to changes in the optical aberrations because of the flexure of the IOL on accommodative effort rather than only on forward movement within the capsular bag.

Except for the forward movement of the IOL, there are accommodative IOLs that are using a different concept. The degree of accommodative effect is related not only to the degree of IOL movement but also to the power of the IOL. If the degree of accommodative shift produced by forward axial displacement of a plus-power lens is proportional to the power of the lens, an accommodating IOL based on the principle of a high-plus-power moving lens coupled to a stationary optically compensatory minus lens should produce higher amplitude accommodative shifts than current single-optic systems based on axial lens displacement [38]. The Synchrony IOL is a dual-optic accommodating IOL consisting of a single-piece, dual-optic, foldable silicone IOL (Synchrony, Visiogen) with an exaggerated high-plus-power moving optic coupled to a low-power static minus lens joined by spring haptic. When implanted in the capsular bag, bag tension compresses the optics, thus reducing their separation. Once accommodative effort arises, the zonules relax, releasing tension on the capsular bag and thus allowing anterior displacement of the anterior optic. In a pilot clinical evaluation by Ossma et al. [55], a mean accommodative range of 3.22 diopters was found in the accommodating IOL group and 1.65 D in the control group with monofocal IOL. Authors conclude that the Synchrony dual-optic IOL shows promise as an option to provide accommodative function in pseudophakic patients. 

Another accommodative IOL with a unique design is the WIOL-CF (A.M.I. Care s.r.o). The WIOL-CF accommodative intraocular lens design is based on the biomimetic principle. According to that principle the hydrogel material used and the lens geometry simulate some of the key properties of the crystalline lens itself. The WIOL-CF can be actually considered more as a natural product and not a typical engineered one. Pseudoaccommodation up to 2 diopters can be achieved with the WIOL-CF. Its soft material and continuous contact with the posterior capsule allows some axial movement and deformation of the lens following ciliary muscle contraction.

## 6. Conclusions

Surgical restoration of accommodation with intraocular implants is a field of modern ophthalmology involving large amounts of investigation and ingenuity. Modern designs of accommodative IOLs are under constant development in order to offer patients independence from spectacles for far and near vision, without comprising visual quality. Many of the currently available accommodative IOLs have already demonstrated favorable results, but it is not yet clear whether they reach the target of restoring real accommodation. Pseudophakic accommodation, that is, the dynamic component of ocular refractive variation during near vision, and pseudophakic pseudoaccommodation, that is, the depth of focus and the subjective adaption to defocus during near vision, are the two core parts of pseudoaccommodation. Currently there is no consensus in the literature on the percentage of the participation of each part in the phenomenon of pseudoaccommodation. Several different methods are utilized by investigators for the study of the phenomenon thus resulting in different results. In spite of that, the concept of pseudophakic accommodation seems to be effectual, although further improvement of the IOL designs is needed.
